# The Prevalence and Severity of Dyspnea in Young Saudi Female Adults: A Cross-sectional Study

**DOI:** 10.2174/0118743064364924250203074821

**Published:** 2025-02-11

**Authors:** Saleh S. Algarni, Majd A. Altamimi, Haifa F. Alqudaimi, Noura M. Aleid, Taha Ismaeil, Hassan Aljohani, Mohammed M. Alqahtani, Mobarak K. Alqahtani, Tareq F. Alotaibi

**Affiliations:** 1 Department of Respiratory Therapy, College of Applied Medical Sciences, King Saud bin Abdulaziz University for Health Sciences, Riyadh, Saudi Arabia; 2 King Abdullah International Medical Research Center, Riyadh, Saudi Arabia; 3 Respiratory Services, King Abdulaziz Medical City, Riyadh, Saudi Arabia

**Keywords:** Dyspnea, Saudi Women, Prevalence, Risk factors, mMRC dyspnea scale, Young Adults

## Abstract

**Background:**

Dyspnea impacts daily activities; women tend to report a higher perception of dyspnea and experience greater emotional distress compared to men. Therefore, the study aims to assess the prevalence and severity of dyspnea among Saudi women aged 18 to 35 years and explore associated risk factors.

**Method:**

A quantitative cross-sectional study was conducted in Saudi Arabia. The data were collected using an electronic online questionnaire survey. The questionnaire consisted of participants’ demographic data, and the status of factors associated with dyspnea, such as body mass index, physical activity, and smoking status. Using the modified Medical Research Council (mMRC) dyspnea scale is another option. The modified Medical Research Council (mMRC) dyspnea scale was used in a descriptive analysis to characterize the prevalence and score of dyspnea.A Mann-Whitney U test and Chi-square tests were conducted to determine the differences and associations according to the dyspnea risk factors. A p-value of <0.05 determined the statistical significance.

**Results:**

A total of 554 participants were recruited. The overall prevalence of dyspnea was reported by 115 (21%) participants, with a mean score of 1.42 (SD 1.38) on the MRC Dyspnea Scale. The majority of participants were from the central region (74%) and aged 18 to 24 years (65.5%). No statistically significant differences were found between participants with and without dyspnea in terms of body mass index (22.6 [19.6-25.6] vs 22.8 [20.1-26.2], p=0.68, respectively), passive smoking status (p=0.07), or physical activity level (p=0.37).

**Conclusion:**

The study concludes that approximately one in five young females experienced dyspnea, suggesting that this symptom may be quite prevalent. However, no significant association was found between dyspnea and factors such as body mass index, passive smoking, or physical activity levels.

## 1 INTRODUCTION

Dyspnea, the distressing sensation of breathlessness, significantly hampers the ability to perform everyday tasks [1]. The causes of dyspnea are numerous. It is particularly common among the elderly, as they are more prone to having lower fitness levels, multiple medical conditions, and reduced daily activity. Obesity and physical inactivity also directly contribute to dyspnea. In individuals with obesity, decreased tidal and residual lung volumes lead to worsening dyspnea and hormonal imbalances [1]. Additionally, both advanced age and a body mass index (BMI) of ≥25 increase mortality risk in cases of severe dyspnea [2].

Gender differences also play a role in dyspnea. While both men and women can experience respiratory diseases, women tend to report a higher perception of breathlessness. Women with dyspnea often experience a lower quality of life compared to men at the same level of breathlessness, possibly due to stronger and more negative emotional responses to the condition [1]. Women are also more likely to experience activity-related dyspnea due to their generally smaller lung volumes [3]. During pregnancy, dyspnea may worsen due to anatomical and physiological changes affecting respiration [4]. Emotions and mood changes have a significant impact on dyspnea. Negative emotions, such as those associated with anxiety and depression, tend to increase the severity of dyspnea and further reduce quality of life. Conversely, positive emotions can alleviate the perception of breathlessness [5].

A study by Currow *et al*. conducted a face-to-face survey in South Australia, assessing persistent dyspnea using a modified Medical Research Council (MRC) dyspnea scale. The survey, which asked respondents about their breathlessness over at least three of the previous six months, found that 8.9% of participants experienced chronic dyspnea. Multivariate analysis revealed that factors such as female gender, part-time or unemployed, low income, and older age were significantly associated with chronic breathlessness [6]. Similarly, Kotlyarov *et al*. employed the MRC scale to quantify dyspnea severity, discovering that both the prevalence and severity of dyspnea increase with age and are more common in women than males. Their additional interviews showed that 6% of respondents attributed their dyspnea to underlying respiratory or cardiovascular conditions [7].

A multicenter study using cross-sectional data from the BOLD study, which included population-based samples from 15 countries, estimated the prevalence of dyspnea and investigated its association with various risk factors such as age, sex, smoking habits, BMI, and spirometry results [8]. The study found that 27% of the 9,484 participants reported experiencing dyspnea. Although the study identified several factors correlated with dyspnea, only 13% of the variation in dyspnea could be explained, and women were found to be twice as likely to report dyspnea as men. Considering that the causes of dyspnea remain unexplained in over 85% of cases, our study aims to describe the prevalence of dyspnea among young adults aged 18 to 35 years and to examine potential differences in associated risk factors.

## 2 MATERIALS AND METHOD

A quantitative cross-sectional study was designed to assess the prevalence and associated factors of dyspnea in Saudi adult females. The target population consisted of Saudi women aged 18 to 35 years, recruited from various regions of Saudi Arabia between May 2023 and December 2023. The study was conducted in accordance with ethical guidelines, and approval was granted by the Institutional Review Board (IRB) of King Abdullah International Medical Research Centre (KAIMRC) under IRB number IRB/1429/23. Informed written consent was obtained from all participants prior to their involvement in the study.

Participants were included in the study if they were Saudi nationals within the specified age range. However, individuals who were active smokers had cardiovascular or respiratory diseases, suffered from chronic diseases affecting respiration, had anemia, had experienced a recent respiratory infection, or had panic attacks were excluded from participation. A total sample size of 554 participants was collected, exceeding the minimum required sample of 385, which was calculated to provide a 95% confidence level with a margin of error of ±5%.

To recruit participants, a non-probability convenience sampling technique was used, with data being gathered through an electronic questionnaire distributed via social media platforms. The questionnaire collected demographic information such as age, marital status, height, weight, income, city of residence, employment status, occupation, and years of service. Additional data on smoking status, physical activity, and body mass index (BMI) were recorded. For participants reporting dyspnea, the severity of breathlessness was assessed using the modified Medical Research Council (mMRC) dyspnea scale. This self-rating tool measures the impact of breathlessness on daily activities. The scale ranges from 0 to 4; grade 0, participants only get breathless with strenuous exercise; grade 1, participants get short of breath when hurrying on level ground or walking up a slight hill; grade 2, on level ground, participants walk slower than people who are in the same age because of breathlessness, or have to stop for breath when walking at their own pace on the level; grade 3, participants stop for breath after walking about 100 yards or after a few minutes on level ground; grade 4, participants are too breathless to leave the house or breathless when dressing/ undressing [9, 10].The questionnaire included a written explanation detailing the mMRC dyspnea scale to ensure understanding and accurate self-assessment.

In Saudi Arabia, Arabic is the official language and is predominantly used for communication by most of the population. The original survey was developed in English, so it was translated into Arabic by an expert in the field. To ensure accuracy, the Arabic version was then backtranslated into English by a different expert. This “forward-backward translation” process, recommended by the World Health Organization, was employed to compare the original and translated English versions. A pilot test was subsequently conducted with 10 members of the public who met the study's inclusion criteria using the Arabic version of the validated questionnaire. The participants reported that the questionnaire was easy to understand and complete. Noteworthy, the pilot study included 10 participants who were selected to reflect the demographic and clinical characteristics of the main study population, ensuring the applicability of the results.

Data collection was facilitated using Google Forms, and the responses were imported into Microsoft Excel before being analyzed with Stata software version 17. Descriptive statistics, including percentages, figures, and tables, were used to represent the demographic data, the incidence rate of dyspnea, and its severity. In addition, a -Mann-Whitney U test and Chi-square test were conducted to determine the differences and association according to the dyspnea risk factors. A p-value of <0.05 determined the statistical significance.

## 3 RESULTS

The study had 554 participants in all. The majority (n=410, 74%) came from the central region, then the western (n=74, 13.5%), eastern (n=33, 6%), southern (n=23, 4%), and northern (n=14, 2.5%) regions.. The distribution of the age showed that most of the participants (n=363, 65.5%) were in the younger subgroup that is between 18 and 24 years old, while (n=191, 34.5%) were between 25 and 34 years old. The marital status revealed that most participants were single (n=444. 80%), (n=98, 18%) were married, and (n=12, 2%) were either divorced or widowed. The median and interquartile range of Body Mass Index (BMI) was 22.7 [20-26]. Regarding the working status, 58% (n=321) were students, 21.5% (n=119) were employed, and 20.5% (n=114) were not employed. Finally, 44% (n=242) of the enrolled participants reported their involvement in performing routine physical activity (Table **1**).

### 3.1 Dyspnea Prevalence and Severity

**Fig. (1):**
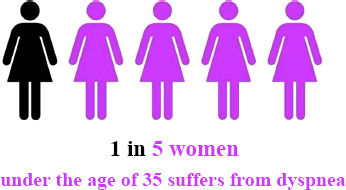
Dyspnea prevalence dyspnea was reported by 115 (21%) of participants. Out of 554 participants

**Fig. (2):**
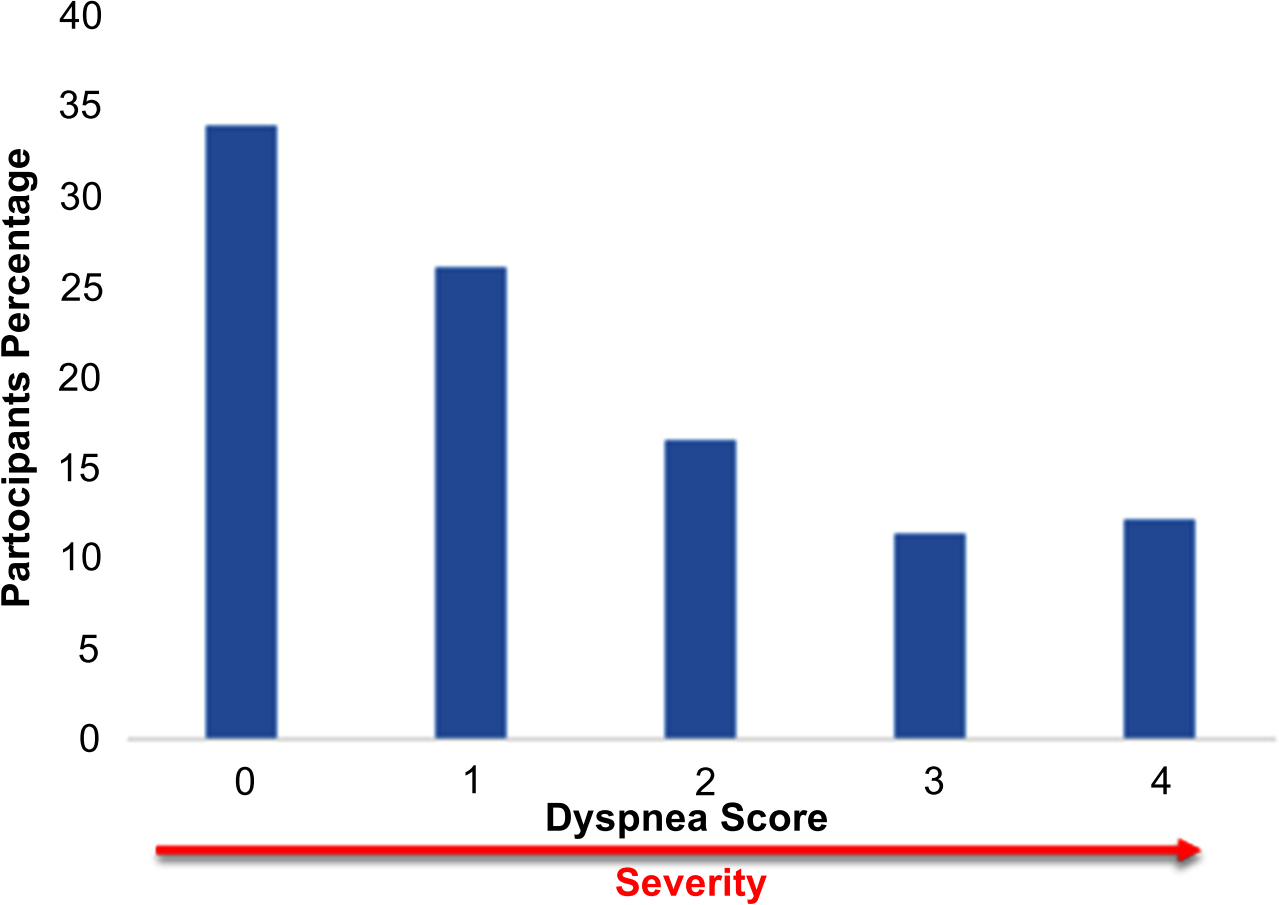
The distribution of dyspnea scores with higher scores representing increasing levels of dyspnea severity and Mean score of 1.42 (SD 1.38) on the mMRC Dyspnea Scale

The prevalence of dyspnea was documented in 115 out of 554 participants (21%), indicating that 1 in 5 Saudi women aged 18 to 35 experiences dyspnea (Fig. **1**). The mean score on the mMRC Dyspnea Scale was 1.42 (SD 1.38), suggesting a relatively mild severity of dyspnea among the participants (Fig. **2**).

### 3.2 Comparison of Participants with and without Reported Dyspnea

Among participants with dyspnea, 70% were aged 18 to 24 years, compared to 64% in the group without dyspnea. Meanwhile, 30% of participants with dyspnea were aged 25 to 34 years, compared to 36% in those without dyspnea. The difference in age distribution between the two groups was not statistically significant (P = 0.21). The median BMI was 22.6 [IQR: 19.6–25.6] in the dyspnea group and 22.8 [IQR: 20.1–26.2] in the group without dyspnea. There was no significant difference in BMI between the two groups (P = 0.68). Passive smoking was reported by 36% of participants with dyspnea compared to 27% of those without dyspnea. This difference approached statistical significance but was not conclusive (P = 0.07). Physical activity was reported by 40% of participants with dyspnea, compared to 45% in the group without dyspnea. This difference was not statistically significant (P = 0.37) (Table **2**).

## 4 DISCUSSION

The presented study aimed to describe the prevalence and severity of dyspnea among women aged between 18 and 35 years in Saudi Arabia. The main findings revealed that dyspnea is a notable health concern, with 21% of the participants reporting it. This indicates that approximately 1 in 5 young Saudi women experience this condition. The mean dyspnea severity score was 1.42, which indicates a relatively mild severity of dyspnea. This implied that while a significant portion of the population reported dyspnea, the symptoms were generally not severe.

A prior multicenter European study that included 9,484 patients from 15 countries found that the estimated prevalence of dyspnea was quite similar [8]. The European study found a higher prevalence of dyspnea in women. Additionally, it was able to identify risk factors that explained dyspnea in only 13% of participants, leaving the causes of dyspnea in the remaining participants unexplained.

The occurrence of dyspnea in a young and healthy population prompts questions about the underlying causes. Several factors, such as environmental factors, lifestyle, and health conditions, could explain this result [2]. In our study, an effort was made to exclude potential contributors, including smoking, respiratory and cardiovascular conditions, chronic illnesses, a recent respiratory infection, panic attacks, and anemia. Participants with these conditions were excluded to ensure that these known factors did not influence the dyspnea observed in the study.

The remaining factors might be related to a high body mass index (BMI), as a previous study has shown that dyspnea is independently associated with weight gain in adults [11]. This relationship is mainly due to decreased lung volume and an increased percentage of fat mass in the central body area [12]. However, it is noteworthy that the median BMI of participants in our study falls within the normal range, indicating that weight gain may not be a major factor in explaining the observed prevalence of dyspnea.

Previous studies have identified exposure to secondhand smoke as a factor that increases the likelihood of experiencing dyspnea [13]. In our research, we assessed participants' exposure to secondhand smoke and found no significant difference between those who reported dyspnea and those who did not. Although no statistically significant finding was observed, the result indicates a borderline significance. One limitation that may have contributed to this is the sample size; a larger sample may provide greater statistical power and help illustrate the association between passive smoking and dyspnea.

The unidentified factors contributing to dyspnea may be connected to air quality. The World Health Organization has recently highlighted the impact of air pollution on respiratory health [14]. Considering the young age of our participants and the lack of any reported chronic conditions, air pollution could be a potential factor linked to dyspnea. However, this is not definitive, and further research is needed to explore the relationship between air quality and dyspnea alongside objective measurements of lung function. Additionally, future research could investigate the link between dyspnea and exposure to strong fragrances. A study by Al Khathlan *et al*. found that 65.7% of Saudi Arabian university students used scented candles, with a significantly higher prevalence among females than males [15]. The most commonly reported health issues associated with scented candle use were coughing, shortness of breath, and headaches. Moreover, future studies might make use of regional or global data on air quality and fragrance exposure in order to place their findings into a wider perspective on their possible impact on the outcomes observed.

In our study, the mMRC Dyspnea Scale was utilized to assess the severity of breathlessness among participants. This scale is a well-validated and reliable tool, which adds strength to our study. Additionally, our focus on young females is notable, as dyspnea is more commonly associated with older age. This research offers preliminary insights that could be valuable for future studies investigating other potential causes of dyspnea. However, there are some limitations to consider. The exclusion of participants with recent infections, panic attacks, or other acute conditions, while this was necessary for the purpose of isolating dyspnea unrelated to these factors, may limit generalization to broader populations in whom such conditions are common. The use of self-reported questionnaires carries the risk of bias, as participants may provide inaccurate or subjective responses.

Additionally, our survey included questions about demographic data, potential factors contributing to dyspnea, and the mMRC Dyspnea Scale. The length and number of questions might have caused participant fatigue, possibly resulting in inaccurate responses. The study’s observational design limits the ability to conclude a causal relationship between measured factors and dyspnea incidence.

The findings of the presented study have considerable implications for both public health and clinical practice. Many young women experience dyspnea, even if not severe, emphasizing the importance of awareness and routine screening to identify and address potential underlying causes early. Additionally, these results highlight the necessity for additional research to study the factors contributing to dyspnea in young women and to develop targeted interventions to reduce its impact on their quality of life.

## CONCLUSION

Dyspnea is a common symptom experienced by nearly one in five young women, highlighting its prevalence among those aged 18 to 35. Despite this high prevalence, the study did not find significant associations between dyspnea and factors such as BMI, smoking, physical activity levels, or other variables. Therefore, further research is essential to uncover the underlying causes of dyspnea and to identify specific demographic groups that may be more vulnerable to this condition.

## AUTHORS’ CONTRIBUTION

It is hereby acknowledged that all authors have accepted responsibility for the manuscript's content and consented to its submission. They have meticulously reviewed all results and unanimously approved the final version of the manuscript.

## Figures and Tables

**Table 1:** Participant characteristics.

Characteristics	n=554	
Regions	Central	410 (74%)
	Northern	14 (2.5%)
	Southern	23 (4%)
	Eastern	33 (6%)
	Western	74 (13.5%)
Age	18 to 24 Years	363 (65.5%)
	25 to 34 Years	191 (34.5%)
Marital status	Single	444 (80%)
	Married	98 (18%)
	Divorced/widowed	12 (2%)
Body mass index, median [IQR]	22.7 [20-26]	
Working Status	Employed	119 (21.5%)
	Not employed	114 (20.5%)
	Students	321 (58%)
Perform routine physical activity	242 (44%)	

**Note: **Data presented as n (%), unless otherwise stated.

**Table 2:** Comparison between participants with and without reported dyspnea.

-		Participants reported dyspnea (n=115)	Participants did not report dyspnea (n=439)	P value
Age	18 to 24 years	81 (70%)	282 (64%)	0.21
	25 to 34 years	34 (30%)	157 (36%)	
Body Mass Index in kg (median [IQR])		22.6 [19.6-25.6]	22.8 [20.1-26.2]	0.68
Passive smoking, n (%)		41 (36%)	119 (27%)	0.07
Performing physical activity, n (%)		46 (40%)	196 (45%)	0.37

## Data Availability

The data and supportive information are available within the article.

## LIST OF ABBREVIATIONS

MRC: = Medical Research Council
BMI: = Body Mass Index

## STANDARDS OF REPORTING

STROBE guidelines were followed.
